# Systolic ShMOLLI myocardial T1-mapping for improved robustness to partial-volume effects and applications in tachyarrhythmias

**DOI:** 10.1186/s12968-015-0182-5

**Published:** 2015-08-28

**Authors:** Vanessa M. Ferreira, Rohan S. Wijesurendra, Alexander Liu, Andreas Greiser, Barbara Casadei, Matthew D. Robson, Stefan Neubauer, Stefan K. Piechnik

**Affiliations:** Division of Cardiovascular Medicine, Radcliffe Department of Medicine, University of Oxford, John Radcliffe Hospital, OX3 9DU Oxford, UK; Siemens Healthcare, Erlangen, Germany

## Abstract

**Background:**

T1-mapping using the Shortened Modified Look-Locker Inversion Recovery (ShMOLLI) technique enables non-invasive assessment of important myocardial tissue characteristics. However, tachyarrhythmia may cause mistriggering and inaccurate T1 estimation. We set out to test whether systolic T1-mapping might overcome this, and whether T1 values or data quality would be significantly different compared to conventional diastolic T1-mapping.

**Methods:**

Native T1 maps were acquired using ShMOLLI at 1.5 T (Magnetom Avanto, Siemens Healthcare) in 10 healthy volunteers (5 male) in sinus rhythm, at varying prescribed trigger delay (TD) times: 0, 50, 100 and 150 ms (all “systolic”), 340 ms (MOLLI TD 500 ms, the conventional TD for ShMOLLI) and also “end diastolic”. T1 maps were also acquired using a shorter readout, to explore the effect of reducing image readout time and sensitivity to systolic motion. The feasibility and image quality of systolic T1-mapping was tested in 15 patients with tachyarrhythmia (*n* = 13 atrial fibrillation, *n* = 2 sinus tachycardia; mean HR range 93–121 bpm).

**Results:**

In healthy volunteers, systolic readout increased the thickness of myocardium compared to the diastolic readout. There was a small overall effect of TD on T1 values (*p* = 0.04), with slightly shorter T1 values in systole compared to diastole (maximum difference 10 ms). While there were apparent gender differences (with no effect of TD on T1 values in males, more marked differences in females, and exaggeration of this effect in thinner myocardial segments in females), dilatation and erosion of contours suggested that the effect of TD on T1 in females was almost entirely due to more partial-volume effects in diastole. All T1 maps were of excellent quality, but systolic TD and shorter readout were associated with less variability in segmental T1 values. In tachycardic patients, systolic acquisitions produced consistently excellent T1 maps (median *R*^2^ = 0.993).

**Conclusions:**

In healthy volunteers, systolic ShMOLLI T1-mapping reduces T1 variability and reports clinically equivalent T1 values to conventional diastolic readout; slightly shorter T1 values in systole are mostly explained by reduced partial-volume effects due to the increase in functional myocardial thickness. In patients with tachyarrhythmia, systolic ShMOLLI T1-mapping is feasible, circumvents mistriggering and produces excellent quality T1 maps. This extends its clinical applicability to challenging rhythms (such as rapid atrial fibrillation) and aids the investigation of thinner myocardial segments. With further validation, systolic T1-mapping may become a new and convenient standard for myocardial T1-mapping.

## Background

Myocardial T1-mapping is a quantitative technique that permits non-invasive assessment of biologically important properties of regional and global myocardium, independent of cardiac function [1]. It relies on alterations of the longitudinal relaxation time constant (T1) of the myocardium due to increased water content or other changes to the local molecular environment [2–7]. Various T1-mapping sequences are available, and these can be broadly divided into inversion recovery methods, saturation recovery methods and mixed inversion and saturation recovery methods [8].

The recently described Shortened Modified Look-Locker Inversion Recovery (ShMOLLI) method uses sequential inversion recovery measurements, permitting a T1 map to be acquired within a short 9-heartbeat single breath-hold [9]. It is a stable and reproducible method for T1-mapping [10], which has already been used in the characterisation of many important cardiac diseases, including acute myocardial infarction [11], acute myocarditis [12], hypertrophic cardiomyopathy [13], dilated cardiomyopathy [13], cardiac amyloidosis [4] and aortic stenosis [14].

The ShMOLLI sequence uses a fixed post-R-wave trigger delay of 340 ms and the resultant trigger time of ~600 ms equates to readout in mid-diastole for typical heart rates (e.g. 60–80 bpm). However, many cardiac diseases are associated with tachyarrhythmia (e.g. atrial fibrillation with fast ventricular response, sinus tachycardia), which can lead to mistriggering and inaccurate T1 estimations. Consequently, the application of inversion recovery T1-mapping methods in cardiac patients with tachyarrhythmias is severely limited.

Our attempts to address this problem during daily clinical imaging led to the hypothesis that reduction in ShMOLLI trigger delay (i.e. “systolic” T1 mapping) may circumvent this issue without significantly affecting estimated T1 values or data quality compared to a conventional diastolic readout. In this study, we systematically altered the ShMOLLI sequence parameters to investigate the effects of systolic T1-mapping on T1 values and data quality in healthy volunteers. We also report initial experience with systolic T1-mapping in patients with tachyarrhythmia.

## Methods

Ethical approval was granted for all study procedures and all subjects gave written informed consent.

### Volunteer experiments

Ten healthy volunteers (50 % male, age 36 ± 7 years) in normal sinus rhythm (HR 61 ± 9 bpm), without symptoms or history of cardiac disease, not on cardiovascular medications and with normal left ventricular function on CMR were included.

#### MR acquisition parameters

All data were collected on a 1.5 Tesla MR system (Magnetom Avanto, Siemens Healthcare, Erlangen, Germany) using a 32-channel cardiac coil array. T1 maps were acquired without administration of any contrast agents based on a prototype ShMOLLI implementation (based on WIP 561, syngo MR B17A version) [9].

**Fig. 1:**
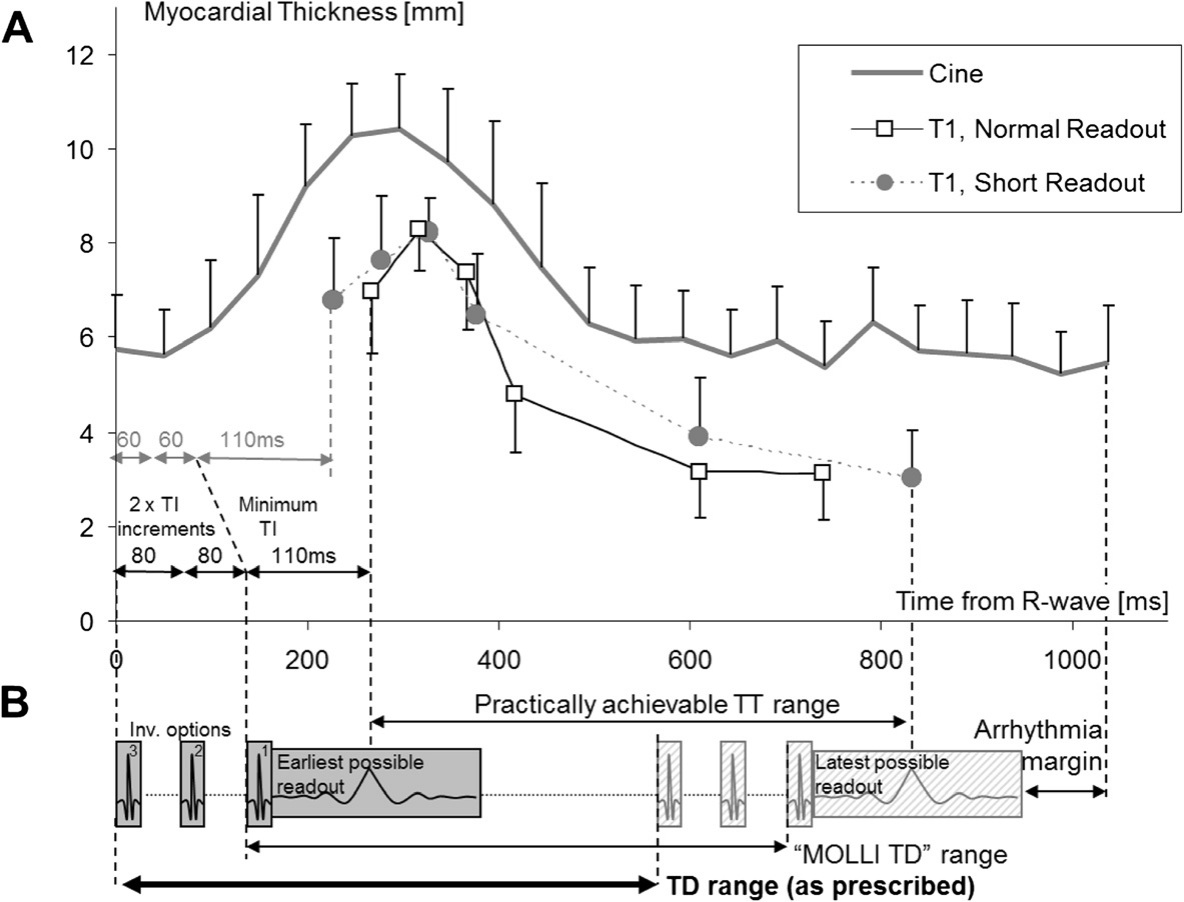
**a** Evolution of myocardial thickness over the cardiac cycle based on cine outlines. Shaded circles and open squares respectively indicate the acquired positions from the R-wave and the estimates of myocardial thickness from the *short* and *normal readout* variants of ShMOLLI studied in this work. Note that myocardial thickness from T1 maps is less by ~1 pixel on each outline (2 mm altogether) to avoid partial-volume impact on T1. **b** Elements of pulse sequence that limit the TD range: the duration of inversion pulse, required delays to achieve the range of inversion times (TI) and readout duration outside of the k-space centre. *Short readout* ShMOLLI achieved earlier TT by reducing TI increments from 80 to 60 ms. The relationship between prescribed TD range, resulting MOLLI TD range and practically achievable TT range is also illustrated

The goal was to alter the image sampling within the cardiac cycle using the permissible range of physiological trigger delay (TD), which, in this WIP implementation, is defined as the time from the R-wave to the start of the inversion preparation in the sequence. Figure 1 illustrates the relation between TD, “MOLLI TD” specific to WIP 561, and the trigger time (TT, time to acquisition of the centre of k-space), such as in cine.

The duration of required preparations limits the shortest MOLLI TD to 270 ms. In order to achieve sampling earlier in the cardiac cycle, we reduced the MOLLI TI increment from 80 to 60 ms [15], which shortened the minimum limit of MOLLI TD to ~230 ms. In order to allow acquisition during higher heart rates (and shorter RR intervals), additional shortening was achieved by using a smaller matrix size of 176 (compared to conventional 192). This reduced the total readout time by approximately ~10 ms. These latter techniques were combined and are henceforth referred to as “*short readout*” (compared to “*normal readout*”). Conversely, the latest readout is limited by the subsequent R-wave. Based on earlier experience we decreased the automatic end-diastolic TD by 50 ms to reduce the occurrence of mistriggering. Full sequence parameters for all combinations tested are provided in the supplementary data.

#### Pilot experiments

A subset of two volunteers underwent a pre-study pilot scan to inform experimental design. Serial T1 maps were acquired with systematic alteration of physiological TD in 50 ms increments from 0 to 500 ms. Based on these data, four “systolic” TDs leading to imaging earlier in the cardiac cycle were chosen for further evaluation: 0, 50, 100 and 150 ms.

#### CMR scan protocol

Following standard planning, end-expiration basal, mid-ventricular and apical short-axis slice positions were selected and used for all T1 maps.

Serial native (or pre-contrast) T1 maps were acquired in a semi-automated fashion using recorded breathing instructions. This minimised variability and ensured that at least 20 s passed between any two T1 maps, to allow sufficient time for full T1 relaxation between experiments.

At each slice position, six different TDs were investigated: the four “systolic” TDs chosen from the pilot experiments (0, 50, 100 and 150 ms), conventional ShMOLLI (340 ms, “MOLLI TD” 500 ms; readout in mid-diastole for typical heart rates of 60–80 bpm) and imaging in “end-diastole” (defined as the captured cycle TD minus 50 ms). For each of these six TDs, two separate T1 maps were acquired: one with the *short readout* parameters for matrix size and TI increment as described above and another with the *normal readout*. In five volunteers, the order of acquisition of the T1 maps was randomised to minimise systematic intra-scan effects (such as change in heart rate or compliance with breathing instructions).

As T1 maps were acquired at 3 slice positions, 6 TDs and with both the *normal* and *short readout*, a total of 36 T1 maps were provided from each volunteer. R^2^ maps (of goodness-of-fit for how well T1 model fitting was achieved for each T1 map) were visually assessed in real time during the scan; acquisitions were repeated if quality was deemed insufficient (as previously described [3]).

### Image post-processing

Offline post-processing was conducted using dedicated in-house software “MC-ROI” written in IDL by SKP (Interactive Data Language, Ver. 6.1, Exelis Visual Information Solutions, Inc., Boulder, USA). Endo- and epicardial contours were manually traced on each T1 map, taking care to avoid partial-volume effects of neighbouring tissues. Each slice was then divided into six segments, using the anterior right-ventricular-left-ventricular insertion point as reference. All voxels in each myocardial segment were analysed, and segmental data extracted for further analysis included mean T1 value, standard deviation of T1 value and median R^2^ value between the manually drawn outlines. No motion correction techniques were employed.

Quality assessment of T1 maps was performed as previously described [3]. The presence of off-resonance artefacts and diaphragmatic movement was assessed by examination of the raw T1-weighted bSSFP images and R^2^ maps by an assessor blinded to TD and *normal*/*short readout*. Segments with suspected artefacts were rejected. Further assessment of quality of T1 maps was provided by analysis of the standard deviation of segmental T1 values; this is a surrogate for T1 map quality in healthy volunteers without known myocardial pathology (which can lead to biological variation in T1 values within segments in patients).

The thickness of myocardium was the average distance between epi- and endocardial outlines towards the centre of the left ventricle. The impact of partial-volume was studied by inflating and eroding the myocardial outlines as previously described [10]. We also tested the myocardial midline values, i.e. a single line of pixels equidistant to endo- and epicardial outlines.

### Validation in patients

In order to examine the feasibility and image quality of systolic ShMOLLI T1-mapping, we tested systolic acquisitions (TD 0 ms–TD 150 ms) in 15 patients with tachyarrhythmia. All patients underwent CMR examinations at 1.5 Tesla (Magnetom Avanto, Siemens Healthcare, Germany) for clinical indications and had a mean HR range between 93 and 121 bpm (*n* = 13 atrial fibrillation, *n* = 2 sinus tachycardia). The degree of tachycardia (resulting in an average shortest RR interval of 488 ms) meant that acquisition using the conventional ShMOLLI sequence parameters would result in mistriggering and it was therefore not possible to directly compare the systolic readout against the conventional sequence in this population without compromising T1 values.

We also present data from a single patient with atrial fibrillation and a slow ventricular response (mean heart rate 40–50 bpm), to demonstrate the occasional need to adjust the TD to optimise T1 fit and improve T1 estimation.

### Statistical analysis

Statistical analyses were performed using IBM SPSS Statistics for Windows, Version 20.0 (IBM Corp., Armonk, NY, USA) and GraphPad Prism version 6 (GraphPad Software, San Diego, California, USA). Normality of data was assessed using the Kolmogorov-Smirnov test. Most variables including T1 and T1 SD did not follow a strictly Gaussian distribution; hence, all statistical tests used were non-parametric. For consistency with other literature reports, we present T1 as mean ± SD; non-parametric data are presented as median and range/IQR.

The Kruskal-Wallis test was used to assess the effect of TD (6 groups); pairwise comparisons were made using the Bonferroni-Dunn approach for variables where the Kruskal-Wallis test was significant. The Kolmogorov-Smirnov test was used to assess effect of *short* versus *normal readout* (2 groups). *P* values < 0.05 (after adjustment for multiple comparisons) were considered significant (*) with smaller P values stratified by size (<0.01 = **, < 0.001 = ***).

## Results

### Volunteer experiments

For each of the 12 combinations of TD and *normal*/*short readout*, the respective maps yielded estimates of 180 myocardial segments (10 volunteers × 3 slices × 6 segments), making a total of 2160 segments collected in the experiment. 54 (2.5 %) segments were excluded due to the presence of suspected image artefacts, leaving 2106 (97.5 %) segments in the final analysis. The variants with short TD (0–50 ms) had the lowest rejection rates (median 1.1 %, range 0.6–1.7 %) and the end-diastolic readout the largest (median 5 %, range 3.9–6.1 %; mostly due to mistriggering). Using the *short readout* resulted in a minor (0.7 %) improvement in segment rejection rates compared to the *normal readout*.

**Fig. 2:**
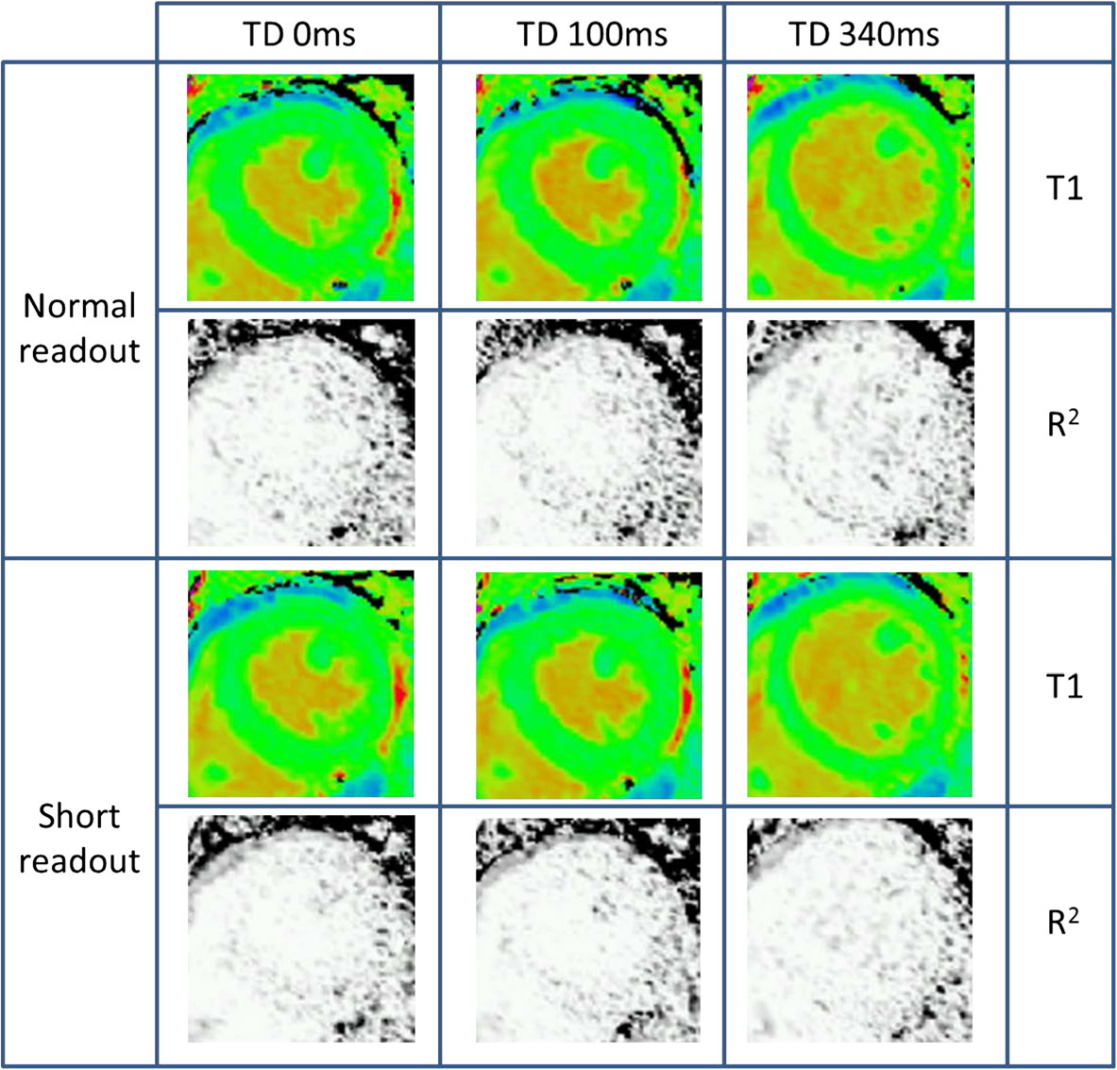
Representative T1 and R^2^ maps acquired from a single volunteer. TD 0 ms, 100 ms and 340 ms (MOLLI TD 500 ms; conventional ShMOLLI) are shown, using both the *normal* and *short readout*. Note the clearly increased myocardial thickness in both systolic acquisitions compared to the conventional diastolic acquisition. T1 values and quality of R^2^ maps are visually similar irrespective of TD and *normal*/*short readout*

All included T1 maps were of excellent quality, with median R^2^ values of at least 0.996. Sample mid-ventricular T1 and R^2^ maps for two systolic TDs and TD 340 ms (standard ShMOLLI) with both *short* and *normal readout* are shown in Fig. 2. The increased myocardial thickness in the systolic TDs compared to the diastolic TD is readily appreciated, but T1 and R^2^ maps have an otherwise similar visual appearance irrespective of the combination of sequence parameters.

#### Effect of TD and normal/short readout on trigger time and myocardial thickness

Across all subjects, the maximal thickness on T1 maps was achieved at TT ~ 320 ms (prescribed TD = 50–100 ms) at 7.5 ± 2.5 mm compared to the standard or end-diastolic acquisitions at 4.7 ± 2 mm (all *p* < 0.001). The variation in trigger time (TT) led to acquisition of T1 maps at different points in the cardiac cycle, with resultant increased myocardial thickness in the systolic acquisitions compared to conventional ShMOLLI or the end-diastolic acquisition.

An example of the evolution of myocardial thickness in representative cine measurements in a single healthy volunteer is shown in Fig. 1, together with the myocardial thickness calculated from the T1 maps. As described in Methods, myocardial outlines on T1 maps were placed away from areas of partial-volume effects; hence the thickness is lower than on corresponding cine images where they are drawn directly on the tissue interfaces. The overlay in Fig. 1 demonstrates the achievable range of cardiac delay times for the MOLLI technique, which is limited by the combined preparation ranges and the arrhythmia range.

#### Effect of TD and normal/short readout on fit quality, T1 values and variability

##### Fit quality

**Fig. 3:**
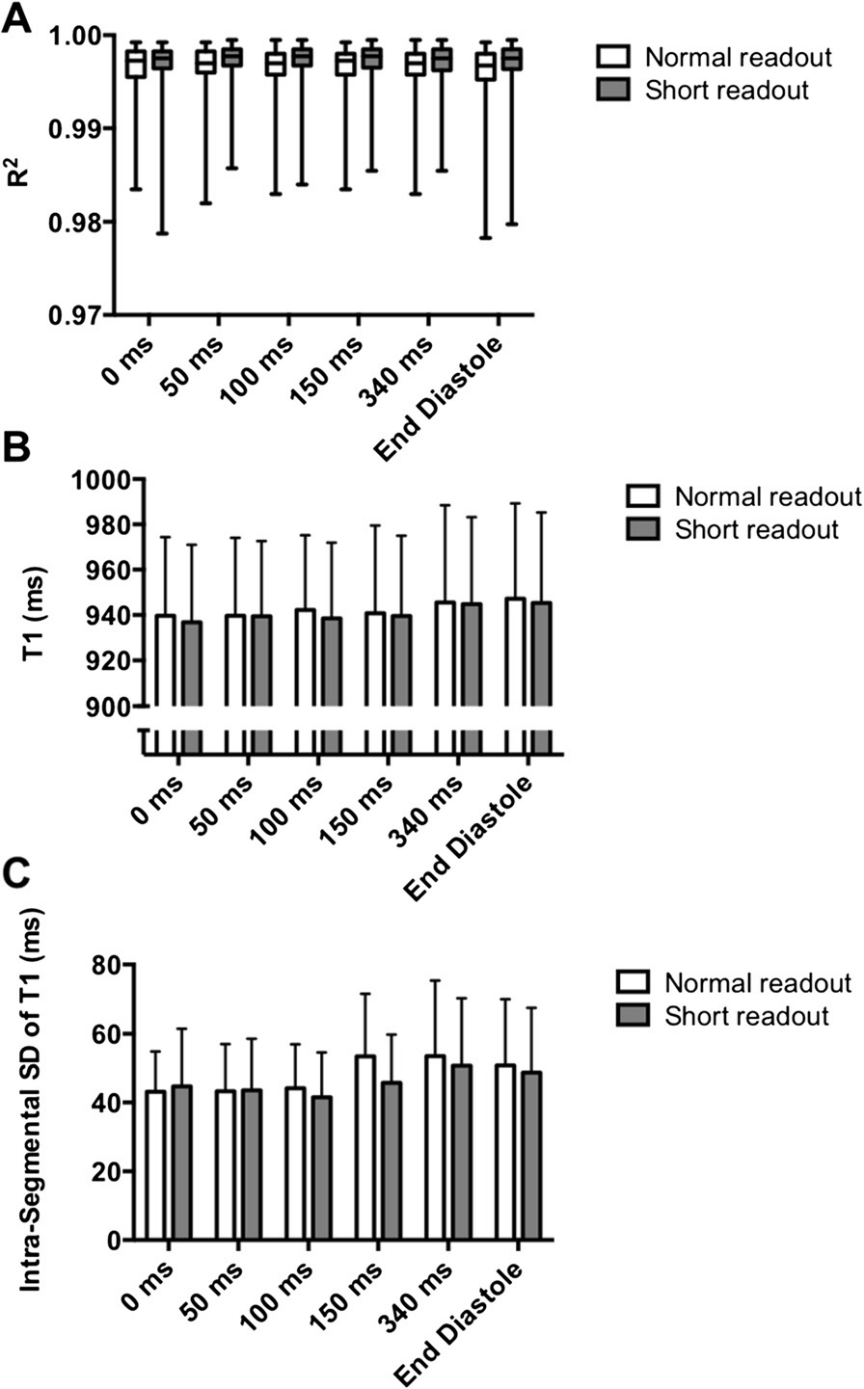
**a** Effect of TD and *normal*/*short readout* on median segmental R^2^ values (median ± range). Note that equivalent R^2^ values were observed irrespective of TD (p=0.36), while higher R^2^ values were obtained with *short readout* compared to *normal readout* (p<0.001). **b** Effect of TD and *normal*/*short readout* on segmental myocardial T1 (mean ± SD). Note slightly shorter T1 values in systole compared to diastole. The apparent effect of *short readout* leading to lower T1 values compared to *normal readout* relates to the slightly shorter TT achieved with the *short readout. P* = 0.04 for overall effect of TD on T1 values. **c** Effect of TD and *normal*/*short readout* on standard deviation of T1 values within each myocardial segment (mean ± SD). Note lower standard deviation in systolic acquisitions (TD < 150 ms) compared to diastolic readout (*p* < 0.001)

TD had no effect on fit quality, as assessed by R^2^ values (*p* = 0.36; Fig. 3a). *Short readout* led to improved R^2^ values compared to *normal readout* (median 0.997 versus 0.996, *p* < 0.001). This improvement was possibly due to increased voxel volume (~19 %) and thus higher SNR, and was independent of TD.

### T1 values

While shorter TD was associated with slightly lower T1 values, with an overall statistical significance (*p* = 0.04), none of the pairwise comparisons of TD reached statistical significance (all *p* ≥ 0.12). Overall significance was lost with minimisation of the partial-volume effects by erosion of myocardial contours (see below). T1 values were not affected by *normal*/*short readout* (*p* = 0.41; Fig. 3b).

### T1 variability

The TD and *normal*/*short readout* had an impact on segmental T1 variability, as measured by standard deviation of segmental T1 values (Fig. 3c). TD < 150 ms was associated with a lower standard deviation of segmental T1 values than TD ≥ 150 ms (mean 43.4 ms versus 50.5 ms, *p* < 0.001). *Short readout* was associated with a lower standard deviation of segmental T1 values than *normal readout* (mean 45.6 ms versus 48.0 ms, *p* = 0.001).

#### Partial-volume effects

Partial-volume effects appear to underlie most of the significant dependencies of T1 values identified above, as revealed by inflation and deflation of the myocardial contours.

**Fig. 4:**
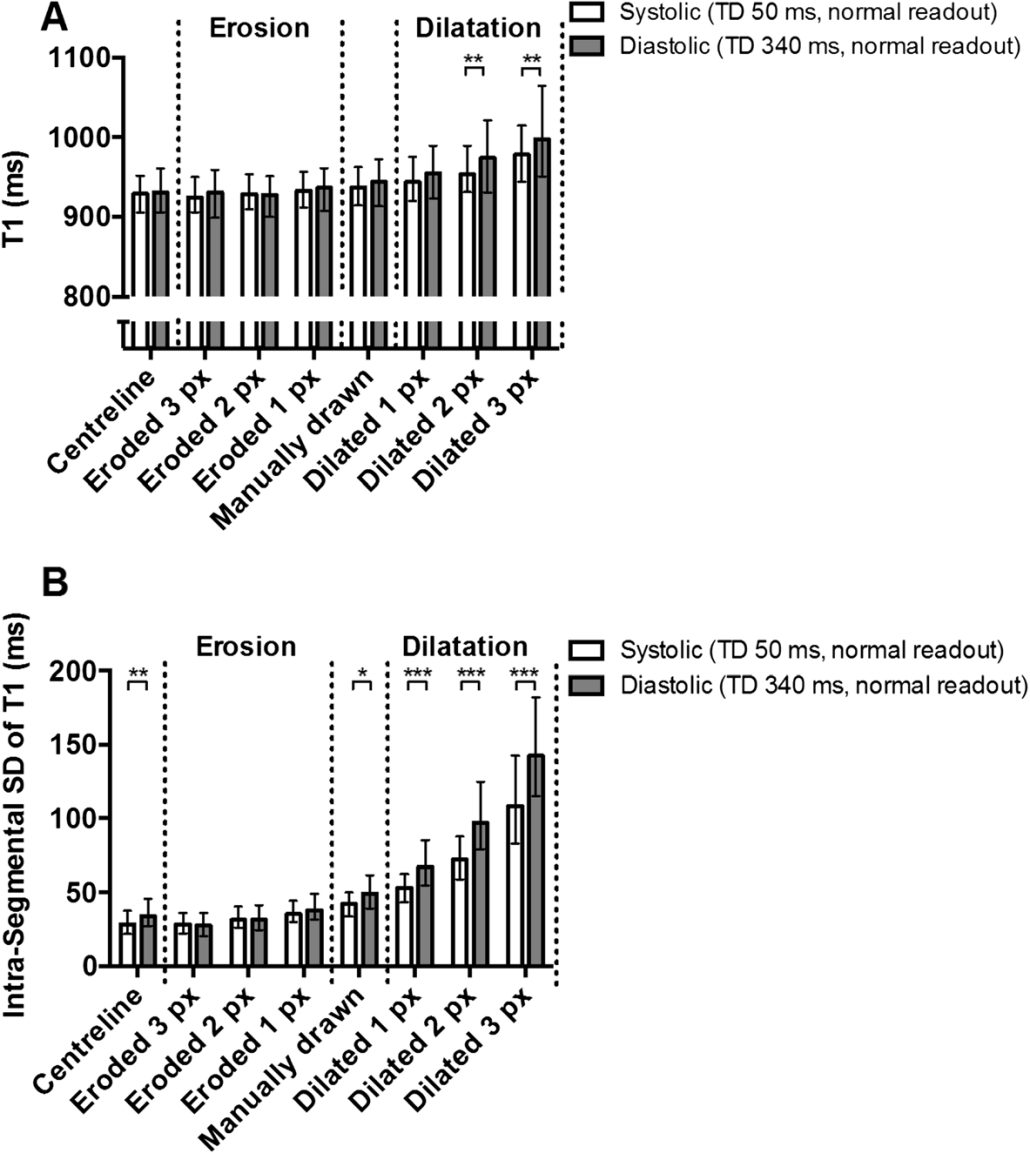
Effect of dilating and eroding manually drawn contours by 1–3 pixels (px) on myocardial T1 (**a**) and intrasegmental T1 SD (**b**) in representative diastolic (TD 340 ms, *normal readout*) and systolic (TD 50 ms, *normal readout*) acquisitions (median ± IQR). Note that dependencies generally decrease with any degree of myocardial volume erosion or with centreline estimates but become amplified when the myocardial outline is inflated, which most affects the thinner myocardium in the diastolic phases. This suggests that partial-volume effects in thinner myocardial segments are responsible for the relationship between TD and myocardial T1 and T1 SD

The identified degree of dependence of T1 on TD (~10 ms, ~1 % of the mean normal T1 value with drawn contours) became smaller with any degree of myocardial erosion or centreline T1 estimates, with no statistical significance (*p* = ns for all). Conversely, the dependence became stronger with any degree of myocardial outline dilatation, and reached statistical significance (*p* ≤ 0.001) for 2 or 3 pixel dilatation when comparing TD 50 ms to conventional diastolic ShMOLLI (TD 340 ms) (Fig. 4a).

Partial-volume effects also appear to underlie most of the effect of TD on intra-segmental pixel T1 variability. Even when the difference is at its strongest comparing the T1 maps acquired with TD = 50 ms versus standard ShMOLLI (smaller pixels, lower SNR and thinner myocardium), the statistical significance is amplified by myocardial contour dilatation but lost for any degree of erosion (Fig. 4b).

#### Effects of gender

**Fig. 5:**
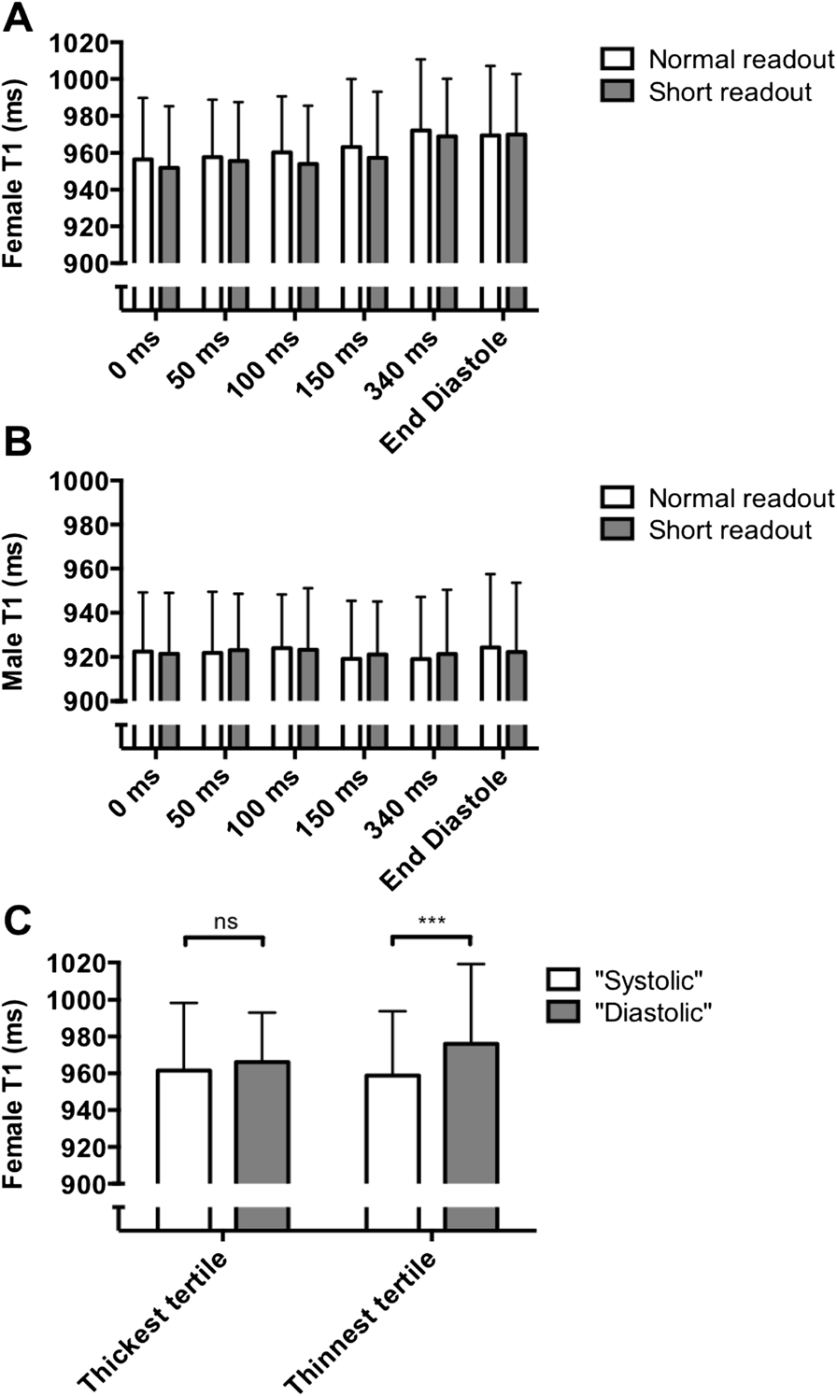
Effect of TD and *normal*/*short readout* on segmental myocardial values in (**a**) females and (**b**) males (mean ± SD). Note the stronger effect of TD on T1 values in females than in the unselected population (see Fig. 3a), and lack of any relationship between TD and T1 values in males. Repeat analysis of segments from females by thickness showed that there was no significant difference in T1 values between diastolic and systolic readout in the thickest tertile (*p* = 0.06), but there was an exaggerated difference for the thinnest tertile (*p* < 0.001) (**c**). This suggests that increased partial-volume effects in diastole (particularly in thinner myocardial segments) are responsible for the gender effect and the relationship between TD and T1 values. Systolic readout was defined as TD 0–150 ms and diastolic readout as TD 340 ms and “end diastole”. The cutoffs for tertiles of female myocardial segmental thickness were defined based on the conventional ShMOLLI sequence thickness in the included female volunteers (<2.97 mm for lowest tertile and > 3.87 mm for highest tertile)

A much stronger dependency of T1 values on TD was noted in females (Fig. 5a) than in males (Fig. 5b), and appears to relate to greater partial-volume effects in females due to thinner myocardium.

In females, diastolic T1 values were a maximum of 20 ms longer than systolic ones (972 ms in diastole versus 952 ms in systole, *p* < 0.001). However in males, there was no significant effect of TD on T1 values, with a maximum difference of 5 ms (924 ms in diastole versus 919 ms in systole, *p* = 0.84).

To ascertain if this was driven by partial-volume effects, we re-analysed the female data for myocardial segments with a thickness in the lowest and highest tertiles of thickness for the female participants (<2.97 mm and >3.87 mm, respectively, when acquired using the conventional 340 ms prescribed TD; Fig. 5c). In the thicker segments, there was no significant difference in T1 values between diastolic and systolic readouts (966 ms versus 962 ms, respectively, *p* = 0.06). In contrast, T1 values were significantly higher with the diastolic compared to the systolic readout in the thinner segments (976 ms versus 959 ms, *p* < 0.001). We infer that the differences seen in T1 values between systolic and diastolic readout in females were driven by greater partial-volume effects in diastole (from adjacent high T1 values in the blood-pool), with thinner myocardium much more susceptible to this phenomenon.

### Validation in patients

In 15 patients with tachyarrhythmia (mean HR range 93–121 bpm), systolic ShMOLLI acquisition parameters produced consistently excellent T1 maps (median *R*^2^ = 0.993). A summary of patient characteristics and T1 and R^2^ results is presented in Table 1.

**Table 1:** Results of systolic ShMOLLI T1 map acquisitions in patients with tachyarrhythmia—either atrial fibrillation (AF) or sinus tachycardia (ST). The majority of acquisitions were undertaken with TD 0 ms (resulting in trigger times ≤ 223 ms) but in five patients a different systolic TD was chosen by the operator. All short-axis systolic T1 maps acquired for each patient are included in this analysis

#	Diagnosis	Rhythm	HR range (bpm)	Number of T1 maps	Mean TT (ms)	Mean T1 (ms)	Median R^2^	Mean RR (ms)	RR range (ms)
1	Rate-related cardiomyopathy	AF	90–140	2	343	943	0.992	683	435–1117
2	Post cardiac arrest	ST	120–130	4	213	1025	0.999	484	462–507
3	Cardiomyopathy	AF	80–120	4	218	975	0.992	645	457–953
4	Takotsubo cardiomyopathy	AF	130–150	2	213	1092	0.995	423	393–520
5	Cardiac amyloidosis	AF	80–100	3	213	1112	0.995	712	570–815
6	Rate-related cardiomyopathy	AF	100–130	1	223	1022	0.994	525	442–600
7	Rate-related cardiomyopathy	AF	90–120	1	263	938	0.993	836	477–1127
8	Rate-related cardiomyopathy	AF	100–120	3	223	984	0.990	668	382–1097
9	Rate-related cardiomyopathy	AF	70–110	2	308	960	0.986	906	567–1495
10	Cardiomyopathy	AF	80–120	7	354	1037	0.990	667	470–1102
11	Post cardiac arrest	AF	90–120	2	223	1035	0.991	762	570–1008
12	Cardiac amyloidosis	AF	70–100	6	218	1142	0.998	742	605–1135
13	Myocarditis	AF	90–120	6	218	981	0.993	732	543–1020
14	Rate-related cardiomyopathy	AF	90–110	2	213	954	0.991	723	472–1025
15	Cardiomyopathy	ST	120–130	3	332	1035	0.997	472	468–475
Average			93–121	3.2	252	1016	0.993	665	488–933

**Fig. 6:**
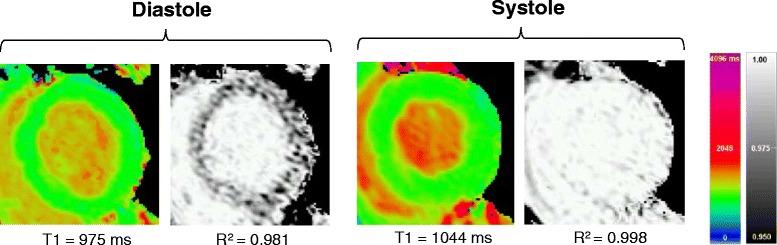
Representative T1 and R^2^ maps acquired using conventional diastolic ShMOLLI T1-mapping (TD 340 ms, *normal readout*) and systolic T1-mapping (TD 0 ms, *short readout*) at 1.5 T. In a patient with sinus tachycardia (mean HR range 120–130 bpm) and myocardial oedema post cardiac arrest, mistriggering of the diastolic T1 map resulted in underestimation of T1 values and distinctively darker appearance of myocardial tissue on the R^2^ map. In contrast, systolic T1-mapping circumvented mistriggering with excellent T1 fit on the R^2^ map. Normal myocardial T1 by ShMOLLI at 1.5 T is 962 ± 25 ms [10]

In contrast, our initial attempts to acquire T1 maps using the conventional sequence parameters (MOLLI TD 500 ms / *normal readout*) resulted in mistriggering in such patients, leading to poor estimation of T1 values. A representative example of this effect is shown in Fig. 6.

## Discussion

In this work, we have demonstrated that alteration of ShMOLLI sequence parameters to permit readout in end-systole (“systolic ShMOLLI T1-mapping”) yields T1 values that are clinically equivalent to those derived by the conventional sequence in healthy volunteers, with the benefit of a slight improvement in data quality. Furthermore, these data suggest that, in healthy volunteers, the T1 differences between diastolic and systolic readout are driven by reduced partial-volume effects in systole due to functionally increased myocardial thickness, implying that the systolic readout may provide a more robust sample of myocardial T1. Finally, we have established that this technique is feasible and produces consistently excellent T1 maps in patients with tachyarrhythmia. To the best of our knowledge, this is the first evaluation and application of a systolic readout inversion recovery method for T1 mapping in tachyarrhythmia, which is highly relevant for real-world clinical applications.

### Variation of T1 values within the cardiac cycle

Our findings of slightly lower T1 values in systole compared to diastole concur in general with some previous reports on the effects of systolic readout using other MOLLI-based sequences [16, 17]. Kawel *et al.* compared a single systolic trigger delay time of 150 ms (“early systole”) with conventional mid- to end-diastolic readout in healthy volunteers, and found diastolic T1 values higher than systolic values, with mean differences of 5 and 15 ms in native T1 values on separate examinations at 3 Tesla (absolute T1 values not reported) [16]. Reiter *et al.* reported an even larger mean difference of 25 ms (984 ms in diastole and 959 ms in systole) [17].

The mechanism for these differences has been attributed to myocardial contraction, which is known to compress the myocardial vascular bed [18]. Reduced intra-myocardial blood volume partition in systole can explain both lower native T1 values (less of longer T1 of blood) and higher systolic myocardial T1 post-contrast (less lower T1 species in blood volume) [16]. While this mechanism is conceptually attractive, ShMOLLI or MOLLI techniques fit T1 values over several heartbeat cycles and would be expected to produce a complex average of their systolic and diastolic blood volume fractions, potentially combined with inflow effects. We are not aware of any theoretical approaches yet that address the relative impact of these underlying effects on myocardial T1.

However, our initial findings [19] have been recently and independently supported by Tessa *et al.* [20], who compare MOLLI T1 maps acquired with a single systolic trigger delay (MOLLI TD 330 ms, shortest possible in Siemens WIP448, which corresponds to TD = 0 ms in this study) against conventional diastolic MOLLI. Similar to us, Tessa et al. found no significant differences between systolic and diastolic T1 values derived from small (4–9 pixels), manually drawn segmental ROIs after extensive removal of segments judged ineligible for analysis. The authors also speculate that the discrepancy between their results (based on a method of analysis that will inherently reduce partial-volume effects) and those of Kawel *et al.* [16] and Reiter *et al.* [17] may emphasise the importance of partial-volume effects in diastole as a factor in the apparent variability of T1 values within the cardiac cycle.

In line with the discussion of future research in Tessa *et al.* [20], we systematically evaluated a number of different systolic and diastolic trigger delays in healthy volunteers with no evidence of cardiac pathology whilst also demonstrating consistently excellent systolic T1 maps in patients with tachyarrhythmia. These data extend confidence in systolic T1-mapping beyond the findings of Tessa *et al.* who studied a heterogeneous group of patients with a variety of myocardial pathologies, including previous myocardial infarction, cardiomyopathy, valve disease, and athletes with echocardiographic or ECG abnormalities. This patient population may have contributed to high rejection rates of myocardial segments in their study, either because they were pathologic on visual evaluation (~7 %) or because they were judged ineligible for analysis (~21 % for diastolic T1 maps) [20]. With the use of inline R^2^ and coloured T1 maps, we achieved a segmental exclusion rate as low as 2.5 %, offering clear diagnostic benefit when assessing pathological T1 changes.

We have also extended the initial investigations of Tessa *et al.* [20] into the importance of partial-volume effects with our systematic manipulation of myocardial contours, examination of differences in T1 between male and female healthy volunteers and exploration of thicker and thinner myocardial segments in females. We conclude that the systole-diastole differences in the presented ShMOLLI experiments can be attributed nearly completely to partial-volume effects in healthy volunteers without known cardiac pathology.

### Potential advantages of systolic T1-mapping and future directions

End systole has a shorter quiescent period than end diastole [21], but is less sensitive to RR variability and arrhythmia. Systolic readout has been advocated for other CMR applications including first-pass myocardial perfusion imaging [22], and has drawn a lot of interest for application in T1-mapping [16, 17, 19, 20, 23]. Our interest in the development and testing of a systolic readout ShMOLLI sequence was to allow reliable T1-mapping in tachyarrhythmia, and our results indicate that this is a valid method for such purposes. The systolic readout naturally increases the myocardial wall thickness and is thus less sensitive to partial-volume effects, which is a certain advantage for a number of additional applications. Our data support the use of systolic T1-mapping in female patients (who have thinner myocardium than males) and these advantages may extend to other situations where partial-volume effects in thin myocardium are a particular issue, such as investigations of apical myocardium or patients with dilated cardiomyopathy, although systematic comparative studies may be warranted.

**Fig. 7:**
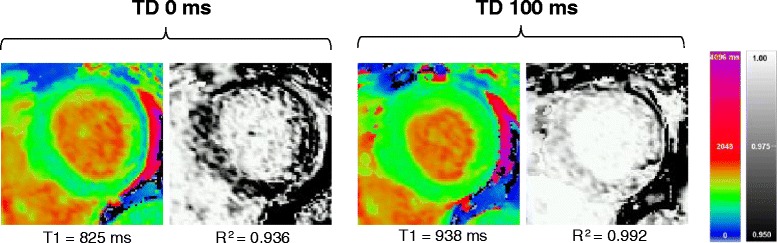
Example of adjustment of TD to optimise T1 fit and improve T1 estimation. In this patient with atrial fibrillation and slow ventricular response (mean HR 40–50 bpm), TD 0 ms (*short readout*) was associated with underestimation of T1 values and distinctively darker appearance of myocardial tissue on the R^2^ map. Examination of cine images led to the recognition that the longer RR interval was associated with delayed peak systolic contraction, and thus TD 0 ms acquired data during the peak of myocardial contraction in mid-systole with associated cardiac motion. Subsequent repeat acquisition with a “tailored” TD of 100 ms (*short readout*) led to data acquisition at end-systole, avoiding myocardial motion and leading to much improved T1 fit on the R^2^ map

Together with findings reported above by other investigators, we are confident that systolic readout offers an important advantage that data can be acquired almost irrespective of heart rate and rhythm; it thus requires little scanner operator experience in handling arrhythmias and can be applied in many clinical situations. Our initial experience of applying the technique in tachyarrhythmia supports this, as the sequence consistently provided T1 maps of excellent quality without need for further adjustment of acquisition parameters. The shortest possible imaging (TD 0 ms) was chosen for simplicity in the majority of these patients, minimising TT and thus maximising heart rate tolerated before mistriggering occurs. The presented healthy volunteer data suggest that a choice of TD 50 ms (corresponding to a TT of 250–300 ms) might serve better to maximise the myocardial thickness. In practice, the only situation in which we have found the choice of TD 0 ms to be suboptimal was in atrial fibrillation with a slow ventricular response. In a patient with such a rhythm (mean HR 40–50 bpm), we noted cardiac motion artefact on raw images, indicating data acquisition during mid-systolic ventricular contraction (rather than the relative lack of cardiac motion characteristic of end-systole). The artefact was easy to identify on R^2^ maps, with the correct timing determined to be TD 100 ms on cine images, and repeat acquisition with this TD resulted in an excellent quality T1 map (Fig. 7).

### Limitations

The number of volunteers included in this study is relatively small, but effects of inter-individual differences are reduced as all combinations of sequence parameters were undertaken in all volunteers. While we tested a specific implementation of ShMOLLI with its corresponding conditional reconstruction, the primary hypothesis has been independently confirmed by another research group using the classic 17-heart-beat MOLLI variant [20]. We focused on validation of systolic ShMOLLI for the determination of native myocardial T1 values. Further studies are needed to identify the implications of the use of this technique for post-contrast T1-mapping and ECV quantification. ShMOLLI acquisitions in this implementation are not motion corrected; while in-plane motion components can be corrected in post-processing, we achieved very low segmental rejection rates in this study without needing to employ this technique. We instead focused on ensuring the best possible breath-holds at time of data acquisition, as this has the added advantage of also reducing the impact of *through-plane* motion. Subjects who have difficulty breath-holding (particularly those with thin myocardial walls) would likely benefit from a combination of both approaches, but this was not specifically tested in this study.

The main limitation for the current evidence in the field is that it is only observational - much further work is needed on simulating the underlying biophysical mechanisms. In particular, it would be hard to extrapolate the current findings to saturation-recovery based techniques, in which end-diastolic readout is usually preferred in order to enable robust saturation time sampling within a single heartbeat. These techniques also do not average T1 recovery over several heartbeats and thus may have different sensitivity to the myocardial blood volumes during the sequence evolution.

## Conclusions

In healthy volunteers, systolic ShMOLLI T1-mapping reduces T1 variability and reports clinically equivalent T1 values to conventional diastolic readout; slightly shorter T1 values in systole are mostly explained by reduced partial-volume effects due to the increase in functional myocardial thickness. In patients with tachyarrhythmia, systolic ShMOLLI T1-mapping is feasible, circumvents mistriggering and produces excellent quality T1-maps. This extends its clinical applicability to challenging rhythms (such as rapid atrial fibrillation) and aids the investigation of thinner myocardial segments. With further validation, systolic T1-mapping may become a new and convenient standard for myocardial T1-mapping.

## Abbreviations

AF: Atrial fibrillation
CMR: Cardiovascular magnetic resonance
IQR: Interquartile range
MOLLI: Modified look-locker inversion recovery
ROI: Region of interest
SD: Standard deviation
ShMOLLI: Shortened Modified Look-Locker Inversion Recovery
SNR: Signal-to-noise ratio
ST: Sinus tachycardia
TD: Trigger delay
TE: Echo time
TI: Inversion time
TR: Repetition time
TT: Trigger time
